# Stokes-Type Entropic Force Driving Nanoscale Phonon-Mediated Superlubricity in 2D Materials

**DOI:** 10.3390/e28101051

**Published:** 2026-09-24

**Authors:** Karol Karpiński, Natalia Kruszewska, J. Miguel Rubi, Adam Gadomski

**Affiliations:** 1 Group of Modeling of Physicochemical Processes, Faculty of Chemical Technology and Engineering, Bydgoszcz University of Science and Technology, Al. Kaliskiego 7, 85-796 Bydgoszcz, Poland; nkruszewska@pbs.edu.pl (N.K.); agad@pbs.edu.pl (A.G.); 2 Departament de Fisica de la Materia Condensada, University of Barcelona, Marti i Franquès 1, 08028 Barcelona, Spain; mrubi@ub.edu

**Keywords:** superlubricity, entropic force, quantum-classical passage for Landauer erasure, Stokes-type dissipation mechanism, fluctuation-dissipation relation, entropy production, phonon generation

## Abstract

This *Communication* addresses a superlubric state in graphene-type layers in nanoscale conditions. We focus on the superlubric state based on the twofold generation and propagation of (thermal) phonons. In the first instance, phonon propagation has a local mode in the graphene-type lattice, whereas in the second instance, it can spread globally. In the former, we see that the kinematic harmonic-mean-type speed condition underlies the mechanism of phonon propagation. In the latter, an arithmetic-mean fingerprint, pointing to a constant acceleration case, can be applied. We disclose a linear phonon-propagation mechanism with an entropic force behind it. Under a passage between classical and quantum domains, this entropic force is equivalent to a Stokes-type force (linear in the sliding speed) with a resultant involvement of a quantum fluctuation–dissipation relation, including a “double quantum” of thermal energy and the apparent (quantum viz. nanoscale) viscosity. The corresponding entropy-production conditions are derived and critically discussed within this framework, establishing a direct thermodynamic link between microscopic (physical) information-processing events and mesoscopic phonon transport. The resulting unified classical–quantum description of entropy-driven phonon-mediated superlubricity constitutes the principal novelty of the present work and may be relevant for future developments in nanotechnology and advanced materials engineering.

## 1. Introduction

### 1.1. Thermodynamics and Information

Reduction in friction-induced energy losses has become one of the major challenges in modern materials science and (nano)technology, driving intense research into ultralow-friction phenomena such as superlubricity. In particular, two-dimensional materials have attracted considerable attention because their layered structures can exhibit extremely low friction under appropriate interfacial conditions [1]. Experimental and theoretical studies have demonstrated superlubricity in graphite and other two-dimensional materials, providing insights into its underlying microscopic mechanisms [1,2,3]. At the nanoscale, however, friction cannot be understood solely in terms of classical mechanical dissipation, because atomic motion, lattice dynamics, and thermal fluctuations become increasingly important. In particular, energy dissipation during sliding can involve the excitation of lattice vibrations, including phonons, providing a microscopic pathway for the conversion of mechanical energy into thermal energy [4]. Understanding the physical limits of energy dissipation at interfaces therefore requires a description that connects mechanical, thermodynamic, and microscopic degrees of freedom.

One approach that provides such a connection is information thermodynamics. Landauer’s principle establishes a minimum energetic cost of $k_{B}T\ln2$ for the irreversible erasure of one bit of information [5,6,7], where $k_{B}$ is the Boltzmann constant and *T* is the absolute temperature. The irreversible erasure of information is accompanied by heat dissipation to the environment, thereby increasing its entropy in accordance with the second law of thermodynamics [6,7]. More recently, information-theoretic concepts based on Landauer’s principle have been applied to nanoscale friction and tribology, including the interpretation of elementary frictional events in terms of information erasure and the derivation of thermodynamic expressions for friction under superlubric conditions [8,9].

The practical implications of these thermodynamic models are particularly relevant to the design and operation of micro- and nanoelectromechanical systems. In these ultrasmall devices, extreme surface-to-volume ratios mean that interfacial friction and stiction can become important contributors to mechanical failure and performance degradation [10]. To probe these interfaces and investigate nanoscale friction, atomic force microscopy (AFM) and friction force microscopy (FFM) serve as key experimental tools. By utilizing AFM with nanometer-scale cantilevers, researchers can measure lateral forces and investigate friction mechanisms and energy-dissipation processes at the atomic scale [10,11].

Furthermore, the overarching dynamics of the sliding system profoundly dictate the magnitude of these energy losses. The relative sliding speed at the interface influences friction through a variety of distinct physical regimes, heavily impacting how energy is dispersed into the surrounding environment, much like in the context of fluid movement [12]. At specific velocity thresholds, the interaction between surface quasiparticles and the sliding body can transition from local stick-slip dissipation to continuous sliding [13]. The stability and lifetime of the superlubric state are also important considerations for practical nanoscale applications, where maintaining ultralow friction over extended sliding distances remains a key challenge [14]. Ultimately, understanding how the derived sliding speeds cause both atomic excitations and interfacial dynamics is essential for engineering surfaces that achieve true superlubricity.

### 1.2. From Classical Friction to Entropy-Driven Superlubricity

The understanding of friction has come a long way, starting from macroscopic empirical observations. In 1699, Guillaume Amontons rediscovered two fundamental laws of friction, originally formulated by Leonardo da Vinci: (a) frictional force is directly proportional to the applied normal load; and (b) the apparent area of contact between bodies does not affect the friction force. A fundamental step toward the microscopic description of friction was provided by Prandtl in 1928, who introduced a model of a point mass moving over a periodic substrate potential while coupled to an elastic restoring force. A closely related molecular theory was subsequently developed by Tomlinson in 1929, and the combined framework is now commonly referred to as the Prandtl–Tomlinson model [15]. This model remains a fundamental “single asperity” approach used today to explain the atomic-scale “stick-slip” phenomenon, which is commonly observed in modern friction force microscopy; for this context, cf. [13,16].

Motivated by these developments, the present work proposes a thermodynamic description of nanoscale superlubricity based on the coupling between Landauer information erasure and the Heisenberg energy–time uncertainty relation. We consider an ultralight AFM scanning probe sliding over a graphene-type surface, both for a pristine interface (Figure 1a) and for an interface containing a van der Waals (vdW) interlayer (Figure 1b). The central assumption is that an elementary sliding event can be associated with the irreversible loss of microscopic positional information and with the corresponding release of thermal energy in the form of phonon excitations. By combining the Landauer energy scale with the Heisenberg relation, we derive an energy-based expression for the coefficient of friction and subsequently examine the resulting sliding velocity, diffusion, and viscosity.

The analysis is restricted to the superlubric regime and does not consider possible adhesion processes. The resulting formulation provides a classical–quantum thermodynamic description of phonon-mediated superlubricity and establishes the basis for the Stokes-type entropic-force interpretation developed in the following sections.

## 2. Models and Methods

### 2.1. Erasure-by-Friction and Sliding Speed

As addressed in [8,9], let us consider an ultralight nanointender sliding over a crystal surface with lattice constant *a*. To this specimen is applied a normal but very tiny load, *W*. It is assumed that displacing the rigid specimen by *Na* (*N* is an integer) crystal-lattice units generates a friction force *F = μW*, following the Coulomb–Amontons law, with *μ* as the (static) coefficient of friction (COF). The work done by the friction force equals the dissipated energy, and it is assumed that all the dissipated energy is fully converted into a “quantum” of (Joule–Lenz) heat, namely ΔQ *=* μWNa.

When activating a frictional interaction of the sample—such as, specifically, a graphene sheet with its corresponding crystal lattice, cf. Figure 1—a certain modification in the microstate’s physical information can emerge. In this situation, the associated work can assume the form of dissipated (Landauer-type) energy *W_x_ = TΔS = TNk_B_ ln*2, where *ΔS* is the entropy change, since we are considering a two-state *N*-atom/unit system at temperature *T*. These derivations use both the first law of thermodynamics, *∆E = ∆Q + W*_x_, where *∆E* is the change in energy, and the Heisenberg energy–time uncertainty principle *∆E∆t~h*, where *∆t* is the characteristic time of an elementary sliding event and *h* is the Planck constant, which leads to the nanoscale coefficient of friction being defined as

(1) $$\mu =\frac{{Nk}_{B}T\;ln2-\frac{h}{\Delta t}}{WNa}.$$

These models can be further extended by the physics of two-dimensional contaminants (adsorbates), which are described using the van der Waals (vdW) equation of state. In such an environment, the contaminants do not hinder superlubricity but may rather undergo adaptation, acting as a virtual lubricating fluid (the so-called “third body” phenomenon). This involves considering modifications of thermal environmental conditions by incorporation of two-dimensional vdW equation of state ${N_{c}k}_{B}T_{mod}=(p-\frac{a_{2}N_{c}^{2}}{A_{0}^{2}})(A_{0}-N_{c}b_{2})$, where $N_{c}$ is the number of contamination particles with two-dimensional vdW parameters $a_{2}$ and $b_{2}$, responsible for interatomic interaction and excluded surface area. The pressure *p* = ${N_{c}k}_{B}T/A_{0}$, with T considered the bath temperature and $A_{0}$ equal to the area of elementary cells interacting with *N_c_* particles. By substituting $k_{B}T_{mod}$ as the thermal energy in Equation (1), the COF is obtained as follows:

(2) $$\mu_{VdW}=\frac{Nln2(\frac{k_{B}T}{A_{0}}+\frac{a_{2}N_{c}}{A_{0}^{2}})(A_{0}-N_{c}b_{2})-\frac{h}{\Delta t}}{WNa}.$$

It is assumed that, during one elementary sliding event, the internal energy remains approximately constant (*dU* ≃ 0), and the Helmholtz free energy, *F*, reduces to the purely entropic contribution *TS*(*x*). The corresponding entropic force follows directly from the free-energy gradient [17]:

(3) $$f_{e}\left(x\right)=-\frac{dF}{dx}=T\frac{dS}{dx}\;.$$

Entropy is given through the Boltzmann relation,

(4) $$S(x)=k_{B}\ln\left(\Omega \left(x\right)\right),$$

where Ω(x) is assumed to be the number of accessible phonon propagation microstates and, within the Landauer framework [7], the elementary sliding process is assumed to generate binary (physical-information) branching of the accessible microstates, yielding $\Omega \left(x\right)=2^{\frac{x}{a}}$. (Outside the binary Landauer framework, the function Ω(x) is generally written as $\Omega (x)=b^{x/a}$, where *b* is the branching factor. For example, choosing b=e (*e* – Napier’s constant) replaces the characteristic ln2 factor in Equation (1) with unity. In this case, the erasure-by-friction mechanism is, unfortunately, not shown. Note that, assuming equiprobable microstates, the probability of a single microstate, labeled *i*, is $p_{i}(x)=1/\Omega (x)=2^{-x/a}$. Then, the Shannon entropy, $S=-k_{B}\sum_{i}p_{i}(x)\ln p_{i}(x),$ reduces to the Boltzmann entropy, $S=k_{B}ln(\Omega (x))$.

Substituting Equation (4) into Equation (3) gives

(5) $$f_{e}\left(x\right)=k_{B}T\frac{d}{dx}\ln\left(\Omega \left(x\right)\right)=\frac{k_{B}Tln2}{a}.$$

This result shows that the entropic force remains constant because the number of accessible phonon-propagation microstates is assumed to increase algebraically “fast” according to the binary Landauer framework. Furthermore, the entropic force vanishes in the limit T→0, consistent with the Nernst–Planck formulation of the third law of thermodynamics.

The characteristic sliding time follows from the superlubricity condition. Because of the superlubrication mode, setting the coefficient of friction to zero in Equation (1),

(6) $$\mu =\frac{Nk_{B}Tln2-\frac{h}{\Delta t}}{WNa}=0,$$

yields the superlubrication-duration time

(7) $$∆t=\frac{h}{Nk_{B}Tln2},\;$$

which is of importance when analyzing in detail the graphene-type systems of interest. This equation reveals an inverse temperature dependence of the characteristic superlubric sliding time, providing the basis for the subsequent definition of the characteristic sliding frequency and the associated phonon transport velocities.

The corresponding elementary sliding velocity *v_SS_* then becomes

(8) $$v_{SS}=\frac{a}{\Delta t}=\frac{a}{h}E_{T}ln2,$$

where $E_{T}=Nk_{B}T$ represents the general thermal energy associated with a sliding event extending over *N* elementary lattice displacements. Equation (8) constitutes the common kinematic basis for both the local harmonic-mean-mechanism and the global arithmetic-mean types of phonon propagation mechanisms unveiled below.

### 2.2. Local Sliding Mechanism

In the present model, “local” denotes the elementary phonon-propagation regime associated with a single lattice displacement between neighboring atomic sites (*N* = 1), whereas “global” denotes propagation over multiple elementary lattice displacements (*N* > 1). In the first case, the elementary sliding event is initiated by the excitation of neighboring atomic pairs. In this regime, the dynamics are governed by the harmonic mean property, and the characteristic thermal energy is simply $E_{T,H}=k_{B}T$, corresponding to the elementary thermal quantum. The elementary sliding velocity is therefore

(9) $$v_{SS}^{\left(H\right)}=\frac{a}{h}k_{B}Tln2.$$

The superscript (H) denotes the use of the harmonic mean.

For the local elementary sliding event, the characteristic transition time is independently recovered from the Heisenberg energy-time uncertainty relation. Indeed, the elementary work performed by the entropic force over one lattice spacing is $∆E=f_{e}a=k_{B}Tln2$, which, together with ∆E∆t=h, leads again to Equation (7) for N=1. Thus, the characteristic sliding time of the local elementary event is independently supported by both the superlubricity condition and the Heisenberg uncertainty principle.

### 2.3. Global Sliding Mechanism

Following a more excessive excitation, the generated phonons propagate collectively over a sliding distance *Na*, corresponding to *N* elementary lattice displacements. This global spreading regime is characterized by the arithmetic-mean property, consistent with a nearly constant acceleration propagation of the excitation. The superlubricity condition first determines the characteristic transition time (Equation (7)), from which the elementary collective sliding velocity (Equation (8)) follows directly. The arithmetic-mean formulation accumulates the contributions of all elementary sliding steps, introducing an additional factor N. Accordingly, the effective thermal energy can be represented as $E_{T,A}=N^{2}k_{B}T,$ leading to

(10) $$v_{SS}^{\left(A\right)}=\frac{a}{h}N^{2}k_{B}Tln2.$$

By analogy with the use of the harmonic mean in Equation (9), the superscript (A) denotes the use of the arithmetic mean.

### 2.4. Quantum Analog of the Stokes-Type Relation

For a local sliding mechanism, comparison of Equation (5) with Equation (9) leads to

(11) $${v_{SS}^{\left(H\right)}={\frac{a}{h}}^{2}\frac{k_{B}T\ln2}{a}=f}_{e}{\frac{a}{h}}^{2},$$

or equivalently

(12) $$f_{e}=\frac{h}{a^{2}}v_{SS}^{\left(H\right)}=\eta_{Q}v_{SS}^{\left(H\right)}.$$

Equation (12) establishes the link between the kinematics of elementary sliding and the concept of an apparent quantum viscosity, suggesting that the macroscopic and dissipative phenomenon of friction resistance emerges from the quantum dynamics of surface energy carriers.

However, the analysis of the phonon propagation mode is treated here as if it were in the local mode; thus, if *N = 1*, an extension for *N > 1* always introduces a *N*-dependence in the analyzed formulae.

The latter expression has the form of a Stokes-type linear-dissipation relation with the apparent quantum viscosity

(13) $$\eta_{Q}=\frac{h}{a^{2}},$$

representing the quantum counterpart of the classical fluctuation–dissipation relation

(14) $$\eta =\frac{E_{T}}{D}=\frac{k_{B}T}{D},$$

where D is the (effective) phonon diffusion coefficient.

The corresponding quantum analog of Equation (14) becomes

(15) $$\eta_{Q}=\frac{h\nu }{D}=\frac{h/\Delta t}{D}.$$

Substituting Equation (13) into Equation (15) yields the phonon diffusion coefficient under superlubric conditions:

(16) $$D=\frac{\frac{h}{\Delta t}}{\frac{h}{a^{2}}}=\frac{a^{2}}{\Delta t}.$$

The obtained expression has the familiar functional dependence on the squared jump length divided by the transition/hopping time, commonly encountered in lattice diffusion and random-walk transport theories. Unlike diffusion coefficients derived from the mean-square displacement of Brownian motion, the present diffusion coefficient follows directly from the fluctuation–dissipation relation applied to entropy-driven phonon transport. Therefore, no prior assumption on the mean-square displacement is involved, and the characteristic numerical prefactor associated with the random-walk process does not enter the derivation. Equation (16) also provides a quantitative realization of this microscopic picture by expressing the effective phonon diffusivity directly through the characteristic transition time Δt, thereby linking elementary sliding, phonon transport, and energy dissipation within a unified thermodynamic framework [18].

Combining Equations (8)–(15) with Equation (5), one obtains

(17) $$D=\frac{a^{2}}{\Delta t^{2}\;}\frac{h}{f_{e}a}=v_{ss}^{2}\;\frac{h}{k_{B}T\;ln2}$$

revealing the square proportionality between the effective phonon diffusion coefficient and the elementary sliding velocity. This establishes a direct connection between phonon transport and the elementary sliding dynamics in the superlubric state. This proportionality is consistent with the general framework of phonon heat transport, in which the phonon propagation velocity is one of the fundamental quantities governing thermal transport [19]. Equation (17) constitutes a direct prediction of the present entropic-force-driven superlubricity model and may be tested by comparing independently determined phonon diffusion coefficients with elementary sliding velocities obtained from atomistic simulations. Moreover, it demonstrates that elementary sliding and phonon transport originate from the same microscopic process, characterized by the lattice spacing *a* and the transition (viz. superlubrication) time Δt, thereby providing a direct and convincing physical connection between the kinematics of elementary superlubric sliding and phonon transport.

The above derivation refers to a pristine graphene-type interface. When a vdW interlayer is introduced between the slider and the substrate, the superlubricity condition is modified through the effective thermal environment described by the two-dimensional van der Waals equation of state. The introduction of a van der Waals interlayer modifies the interface through which phonons propagate. Since an interface represents an interruption of the crystalline lattice supporting phonon transport, the thermodynamic conditions governing superlubricity are correspondingly altered [19]. Thus, the characteristic transition time obtained by setting $\mu_{VdW}=0$ in Equation (2) differs from that of the pristine interface (cf. Equation (6)), yielding the modified local sliding velocity

(18) $$v_{SS}^{vdW}=\frac{ak_{B}T\left(1+\frac{a_{2}N_{c}}{A_{0}k_{B}T}\right)\left(1-\frac{N_{c}b_{2}}{A_{0}}\right)ln2}{h}.$$

Following the same derivation as for the pristine interface, the effective diffusion coefficient in the presence of a van der Waals interlayer becomes

(19) $$D_{vdW}=\frac{a^{2}}{\Delta t}\frac{\left(1-\frac{N_{c}b_{2}}{A_{0}}\right)}{1-\frac{{\ln2\;a}_{2}N_{c}\Delta t\left(1-\frac{N_{c}b_{2}}{A_{0}}\right)}{hA_{0}}},\;or\;D_{vdW}=\frac{a^{2}k_{B}T{\left(1+\frac{a_{2}N_{c}}{A_{0}k_{B}T}\right)}^{2}{\left(1-\frac{N_{c}b_{2}}{A_{0}}\right)}^{2}ln2}{h}$$

Unlike Equation (17), the effective diffusion coefficient explicitly depends on temperature owing to the attractive ($a_{2}$) and excluded-area ($b_{2}$) corrections introduced by the two-dimensional van der Waals equation of state. The vdW interlayer therefore modifies the elementary phonon transport by changing the thermodynamic conditions under which the superlubric state is established. Moreover, the presence of the vdW interlayer modifies not only the coefficient of friction but also the characteristic sliding velocity and the effective phonon diffusion coefficient, thereby providing a unified thermodynamic description of contaminant-assisted superlubricity.

### 2.5. Entropy Production

Within the framework of mesoscopic nonequilibrium thermodynamics (MNET) [20,21], the local entropy-production rate is expressed as the product of a generalized flux and its conjugate thermodynamic force,

(20) σ=JX.

For the sliding coordinate x, the generalized flux is identified with the steady sliding velocity, $J=\frac{dx}{dt}=v_{SS}$, and the corresponding thermodynamic force (being, in general, not a Newtonian force) is obtained from the gradient of the free energy, $X=\;-\frac{1}{T}\frac{\partial F}{\partial x}=\frac{f_{e}}{T}$ (cf. Equation (3)), where *F* denotes the Helmholtz free energy. Such an approach is consistent with the mesoscopic description of phonon transport, where heat transport is directly related to entropy flow through quantum channels [22], as well as with information-theoretic descriptions of nonequilibrium transport emphasizing the evolution of entropy in diffusion processes [23].

Substituting the above expressions into Equation (20) gives

(21) $$\sigma =\frac{v_{SS}f_{e}}{T},$$

which, together with Equations (5) and (8), yields the local entropy-production rate associated with the elementary sliding event

(22) $$\sigma =\frac{k_{B}^{2}{\left(ln2\right)}^{2}T}{h},$$

or, using the proportionality of Δt and T (see Equation (7)), $\sigma =\frac{k_{B}ln2}{\Delta t}$.

Equation (22) shows that the entropy-production rate is directly proportional to the temperature. Remarkably, the proportionality coefficient depends exclusively on the fundamental constants $k_{B}$ and *h*, together with the characteristic Landauer two-state factor, ln2, while all explicit geometric quantities, such as the lattice spacing a, are cancelled in phonon propagation. Finally, σ→0 as T→0, which is consistent with the disappearance of thermal phonon excitations and the corresponding suppression of dissipative processes in the zero-temperature limit. This linear temperature dependence of the entropy-production rate is consistent with mesoscopic phonon transport, where the transported quantity is thermal energy (equivalently, entropy), leading to a thermal conductance proportional to temperature in the ballistic limit [22].

An independent derivation of the same entropy-production rate can be obtained by comparing the present energy balance with the nonequilibrium formulation of the second law of thermodynamics proposed by Esposito and Van den Broeck, since Equation (1) exhibits the same energy-balance structure as their fundamental relation [7]:

(23) $$T\Delta_{i}S=W_{x}-\Delta F,$$

where $\Delta_{i}S$ is the irreversible entropy production and ΔF is the change in nonequilibrium free energy.

Indeed, per Equation (1),

(24) $$\mu WNa={Nk}_{B}T\;ln2-\frac{h}{\Delta t}$$

can be interpreted term by term by identifying $T\Delta_{i}S=\mu WNa,$ representing the irreversible energy dissipated by friction, $W_{x}=Nk_{B}Tln2,$ representing the total available Landauer energy, and $\Delta F=\frac{h}{\Delta t}$ as the free-energy contribution associated with the elementary phonon excitation. The latter allows the entropic force to be expressed as

(25) $$f_{e}=-\frac{\partial \Delta F}{\partial x}=-\frac{\partial \Delta F}{\partial ∆t}\cdot \frac{\partial ∆t}{\partial x}=\frac{h}{{∆t}^{2}}\cdot v_{SS}^{-1}.$$

Substituting this result into Equation (21) gives

(26) $$\sigma =\frac{v_{SS}f_{e}}{T}=\frac{h}{{T∆t}^{2}}.$$

Using the local superlubricity condition established in Section 2.2,$\;k_{B}Tln2=\frac{h}{∆t}$, together with the entropy-production rate given by Equation (22), one can obtain

(27) $$\sigma =\frac{k_{B}^{2}{\left(ln2\right)}^{2}T}{h}=\frac{{\left(\frac{h}{T∆t}\right)}^{2}T}{h}=\frac{h}{{T∆t}^{2}},$$

which is identical to Equation (26). Hence, the entropy-production rate is obtained independently from two complementary routes: (i) the MNET flux–force formulation and (ii) the nonequilibrium free-energy representation based on the second law of thermodynamics and the Landauer–Heisenberg relation.

For T=100 K and the corresponding characteristic superlubric transition time $\Delta t=6.9238\times 10^{-13}$ s, the present model predicts an entropy-production rate of $1.3822\times 10^{-11}\;J\;K^{-1}\;s^{-1}$. Although direct quantitative comparison with the literature is difficult because entropy-production rates depend on the specific nonequilibrium model and experimental configuration, the obtained value is consistent with the extremely weak irreversible dissipation expected for phonon-mediated superlubric transport at the nanoscale. This interpretation is further supported by stochastic thermodynamic descriptions of nanoscale friction, where entropy production is identified as a direct measure of the irreversible heat generated during sliding, as well as by experimental demonstrations that thermal energy at low temperatures is transported through quantized phonon channels [22,24].

The entropy-production rate derived above also provides a natural starting point for deriving an evolution equation for the local surface density of Landauer-associated phonon excitations. Accordingly, the entropy-production rate naturally appears as the source term in the local entropy balance equation

(28) $$\frac{\partial s(r,t)}{\partial t}+\nabla \cdot J_{s}(r,t)=\sigma (r,t),$$

where *s*(*r,t*) denotes the local entropy density, *r* is the position vector on the graphene interface, and $J_{s}(r,t)$ is the entropy flux [20,25].

For the two-dimensional graphene interface considered here, the entropy balance is formulated per unit surface area. Accordingly, the entropy-production term σ(r,t) represents the local entropy-production rate per unit area, rather than the total entropy-production rate σ given by Equation (22) and associated with a single elementary sliding event, i.e., $\sigma \left(r,t\right)=\frac{\sigma }{A_{e}}$, where $A_{e}$ denotes the characteristic area associated with an elementary sliding event (e.g., $A_{e}\approx a^{2}$).

Therefore, the present model predicts that the irreversible entropy generated during an elementary sliding event acts as the local source term in the entropy balance, while its subsequent redistribution is governed by the entropy flux. To derive an evolution equation for the density within the present model, we introduce $n_{L}(r,t)$ as the local surface density of Landauer-associated phonon excitations, i.e., the number of elementary Landauer events per unit area. Since each elementary Landauer event carries the entropy increment $\Delta S_{L}=k_{B}ln2$, the local entropy density can be written as

(29) $$s\left(r,t\right)=k_{B}\ln2\;n_{L}\left(r,t\right),$$

and accordingly, the entropy flux may be expressed as

(30) $$J_{s}(r,t)=k_{B}ln2\;J_{L}(r,t),$$

where $J_{L}(r,t)$ denotes the flux of these effective excitations. The substitution of these relations into the local entropy balance equation (Equation (28)) yields

(31) $$\frac{\partial n_{L}}{\partial t}+\nabla \cdot J_{L}=\frac{\frac{\sigma }{A_{e}}}{k_{B}\ln2}.$$

Since σ(r,t) is defined per unit area, the right-hand side has the units $m^{-2}\;s^{-1}$, corresponding to the local generation rate of Landauer-associated phonon excitations.

Upon substituting the entropy-production rate predicted by the present model (Equation (22)) and using $\Delta t=\frac{h}{k_{B}T\ln2}$ (cf. Section 2.2), the density-evolution equation becomes

(32) $$\frac{\partial n_{L}}{\partial t}+\nabla \cdot J_{L}=\frac{\sigma }{k_{B}\ln2A_{e}}=\frac{k_{B}Tln2}{hA_{e}}=\frac{1}{∆tA_{e}}.$$

The right-hand side therefore represents the local generation rate of effective Landauer-associated phonon excitations per unit interfacial area. Within the present model, this corresponds to the generation of one elementary excitation over the characteristic area $A_{e}\approx a^{2}$ during the characteristic transition time Δt.

Thus, the entropy-production term determines the local generation rate of Landauer-associated phonon excitations, while the flux term governs their subsequent transport across the graphene interface. Moreover, Equation (32) provides a mesoscopic density-evolution equation that links the irreversible entropy generated during elementary sliding events with the transport of the corresponding effective excitations.

## 3. Results

In this section, the theoretical relationships derived in Equations (9) and (16)–(18) were used to analytically calculate the sliding velocity and phonon diffusion coefficient for a graphene monolayer with a lattice constant of 0.246 nm, as illustrated in Figure 1. By adjusting the model to appropriate structural and energetic data (the lattice constant, elementary surface area, suitable binding energy as associated with the corresponding vdW parameters [26], etc.) the approach can be applied, for example, to hexagonal boron nitride (h-BN) or transition metal dichalcogenides (TMDs). The parameters governing the two-dimensional vdW equation of state were chosen to represent a water contaminant layer, defined by the constants $a_{2}=1.96\times 10^{-39}\;J\;m^{2}$ and $b_{2}=0.1310\;nm^{2}$. The elementary surface area was set to $A_{0}=0.1665\;nm^{2}$.

Figure 2 shows the temperature dependence of the elementary sliding velocity for clean and contaminated graphene interfaces. The superlubric sliding speed increases linearly with temperature in both cases, reflecting the proportionality $v_{SS}\propto E_{T}$. However, the contaminated system demonstrates a distinctly smaller slope alongside a higher initial speed at lower temperatures. Conversely, the clean system starts from a lower intercept but rises much more steeply.

This behavior suggests that the contaminant layer partially screens the coupling between thermal excitation and sliding kinematics, which may be associated with enhanced phonon scattering and viscous damping within the interfacial layer. The higher low-temperature speed in the contaminated case can be attributed to the intrinsic molecular mobility within the intercalated water layer, which provides an initial lubricating effect at the baseline. However, the reduced slope suggests that at higher temperatures, this contaminant layer acts as a thermal and kinematic buffer. It actively screens the direct coupling between the temperature gradient and the sliding kinematics, likely by increasing inelastic phonon scattering and acting as a viscous damper.

These results can be qualitatively compared with recent molecular-dynamics simulations of water-intercalated graphene interfaces [27]. As shown in Figure 2, the introduction of the interfacial layer increases the characteristic sliding velocity relative to the pristine interface at low and intermediate temperatures, with the difference diminishing toward the crossover temperature. This enhanced sliding mobility is qualitatively consistent with the reduced frictional energy dissipation reported by Li et al. [27] for water-intercalated graphene. However, this comparison should be regarded as qualitative, since the present $v_{SS}$ represents the characteristic velocity of an elementary sliding event, whereas Li et al. evaluated energy dissipation directly from atomistic simulations.

Figure 3 compares the diffusion coefficients as a function of lubrication time (a) and temperature (b). Both models exhibit a rapid, monotonic decay of the diffusion coefficient as the time scale increases. Notably, the curve representing the non-contaminated, phonon-mediated system remains consistently above the local reference curve throughout the observed range. Under superlubric conditions $\left(\mu_{VdW}=0\right)$, lubrication time and temperature are directly related through Equation (7); the time is limited by the T > 0 condition. This time limit is equal to $\Delta t_{T\to 0}=\frac{hA_{0}}{ln2\;a_{2}N_{c}\left(1-\frac{N_{c}b_{2}}{A_{0}}\right)}$. With our data, this corresponds to $\Delta t_{T\to 0}=3.809\times 10^{-13}$ s.

These results indicate that mass and momentum transport are highly efficient at ultra-short timescales but quickly relax toward negligible values. Such behavior is characteristic of transient, phonon-assisted motion, which relies on initial phase coherence that rapidly dissipates due to thermalization and environmental damping over longer periods. The elevated diffusion curve in the non-contaminated system suggests that the vdW-mediated collective interactions within the water layer sustain this short-term transport regime less effectively, temporarily delaying the onset of complete decoherence compared to the strictly local mechanism. As Δ*t* approaches Δ*t*_(T→0)_, the diffusion coefficient of the contaminated system increases sharply. This behavior may be attributed to the increasing influence of the interfacial layer, which effectively smoothens the potential-energy landscape and facilitates particle motion. It should be emphasized that this thermodynamic condition originates from temperature variations induced by particles within the interfacial layer.

Figure 2 and Figure 3a,b exhibit a crossover point at which the contaminated and non-contaminated sliding velocities and diffusion coefficients become identical. This value corresponds to temperature $T=\frac{a_{2}(1-\frac{N_{c}b_{2}}{A_{0}})}{k_{B}b_{2}}=231.06K$. This crossover occurs when the contributions of intermolecular interactions and excluded surface area compensate each other. This condition is connected to time $∆t=2.997\times 10^{-13}$ s. The time range between this time and $\Delta t_{T\to 0}$ are connected to larger-than-local diffusion and sliding speed. Outside this range, the impact of excluded surface area is decisive.

As one can see in Figure 3b, paradoxically, the data reveals an inverse relationship: diffusion is notably higher at lower temperatures.

This counterintuitive phenomenon can be understood in terms of the interplay between thermal fluctuations, phonon scattering, and the structural organization of the contaminant layer. At ultra-low temperatures, thermal noise is minimal, allowing for highly coherent, ballistic-like phonon propagation. Furthermore, the localized water molecules may adopt a more ordered, rigid (ice-like) interfacial structure that provides a smoother and more uniform potential-energy landscape for sliding. As the temperature rises, increasing thermal energy is expected to disrupt this ordered state and may enhance phonon scattering, including Umklapp processes. Consequently, a larger fraction of the kinetic energy associated with sliding is dissipated through entropy production. The increased thermal excitation may also promote additional scattering and localization of phonons within the increasingly disordered contaminant layer, thereby reducing the diffusion coefficient and suppressing coherent transport.

## 4. Conclusions and Perspectives

In this study, we propose a mechanism that provides a consistent classical–quantum thermodynamic framework capable of sustaining the superlubricity regime in rubbing nanoscale systems. Within this framework, complementary harmonic- and arithmetic-mean sliding velocities naturally emerge, governing the local and global phonon-propagation regimes, respectively. Their unified description further demonstrates that the resulting entropic driving force admits a natural Stokes-type dissipative interpretation through the concept of an apparent quantum viscosity. Consequently, the present results identify a Stokes-type entropic force as the thermodynamic mechanism driving phonon-mediated superlubricity in graphene-type interfaces.

Operating at the classical–quantum interface, this framework consistently combines equilibrium thermodynamic principles with the linear nonequilibrium flux–force formalism of Stokes–Onsager, thereby describing the linear dissipation of mechanical frictional energy into discrete quanta of thermal energy.

Consistent with the proposed thermodynamic picture, the associated entropy-production rate, evaluated independently by two equivalent theoretical approaches and interpreted within the same linear-dissipation (Stokes-type) framework, remains extremely small under the low-temperature conditions considered. Therefore, the derived expression for the entropy-production rate, σ, establishes a direct thermodynamic link between the microscopic Landauer–Heisenberg description of elementary sliding events and the mesoscopic entropy balance governing irreversible phonon-mediated energy transport.

Building upon this thermodynamic connection, the proposed framework naturally yields a mesoscopic evolution equation for the local surface density of Landauer-associated phonon excitations, thereby providing a dynamic description of the generation and transport of these effective excitations at the interface.

More broadly, the present results are consistent with previous information-theoretic studies of nonequilibrium diffusion, which have highlighted the interplay between entropy evolution, transport processes, and localization [16].

It is worth noting that the H-case (of the harmonic mean) for the quasiparticles’ propagation might open space for further considerations, for example, ones based on the Unruh effect, in cases where the rubbing system takes place in highly demanding high-acceleration astrophysical conditions [28].

Taken together, these findings open a route toward a unified thermodynamic description of superlubricity in which microscopic (physical) information processing, entropy production, and collective interfacial transport emerge as complementary aspects of the same irreversible phenomenon.

Presumably, an interesting extension of the present theory would be to incorporate stochastic fluctuations into the elementary sliding process. In particular, it would be worthwhile to investigate whether the Landauer–Heisenberg description of elementary sliding events can be interpreted within the framework of fluctuation relations, such as the Jarzynski equality (see Equation (18) in Ref. [17]; for a critical assessment of its direct application to friction force microscopy, also see [29]), while accounting for their known domains of validity and limitations in systems driven out of equilibrium [30]. Such an extension could provide new insights into the transition from equilibrium to nonequilibrium thermodynamics in entropy-driven superlubricity.

Overall, we present here a simple theoretical viz. analytic model, the novelty of which relies on its suitability for disclosing the friction-lubrication effect at the nanoscale, the scale naturally equipped with the classical-quantum ‘paradigmatic passage’ uncovered by the current study with the purpose of supporting the nanotechnology of superlubric *2D* materials, no matter whether pristine or decorated with contamination layers. The model refers, whenever appropriate, to other studies, such as those based on MD (ab initio), DFT etc., but any straightforward connection of our modeling to its simulation-based counterparts has so far been revealed by fairly similar trends when analyzing the superlubric behavior, albeit not formally justified or strictly verified. However, some data from graphene-involving experiments have been included solely when presenting the results. Otherwise, non-graphene two-dimensional rubbing systems have not been touched upon but are plausible for an analysis by changing, for example, the structure (surface-design) parameters of the support, etc.

## Figures and Tables

**Figure 1:**
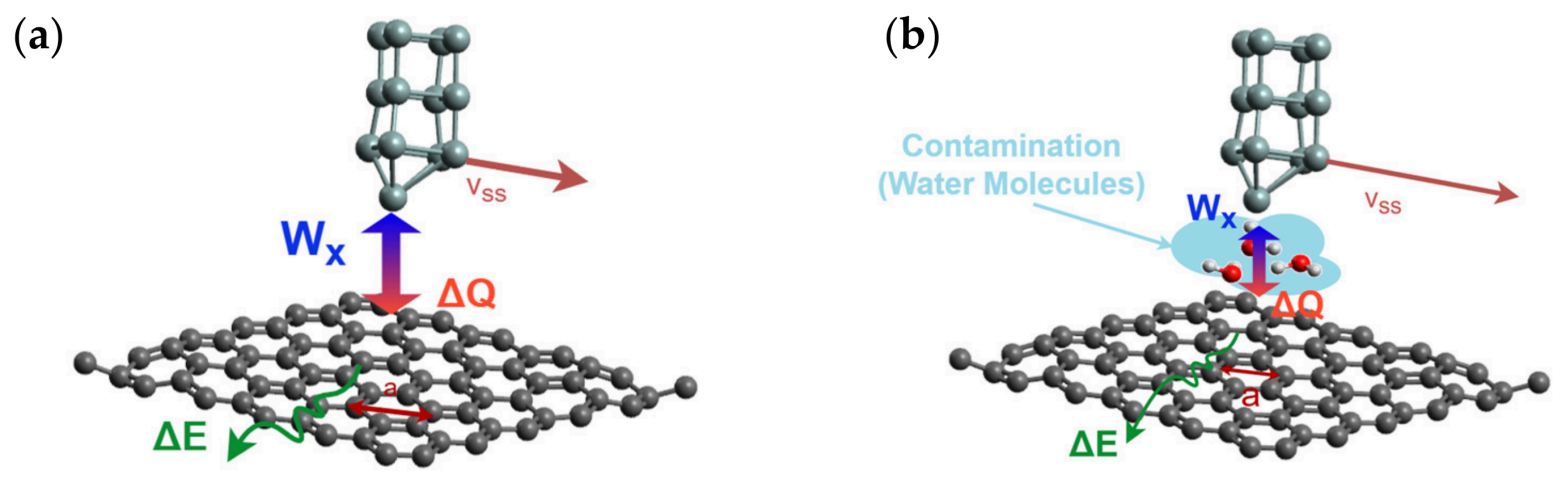
Schematic representation of a graphene surface interacting with a sliding body moving at the elementary sliding velocity $v_{SS}=\frac{a}{\Delta t}$, where Δt denotes the characteristic time of an elementary sliding event: (**a**) pristine interface and (**b**) interface containing a van der Waals (vdW) interlayer. The lattice constant is denoted by a. The frictional work associated with defect formation is assumed to be entirely dissipated as heat in accordance with the second law of thermodynamics. The free-energy contribution is described through the Heisenberg energy–time uncertainty relation, providing the quantum counterpart of the thermodynamic energy balance [8,9].

**Figure 2:**
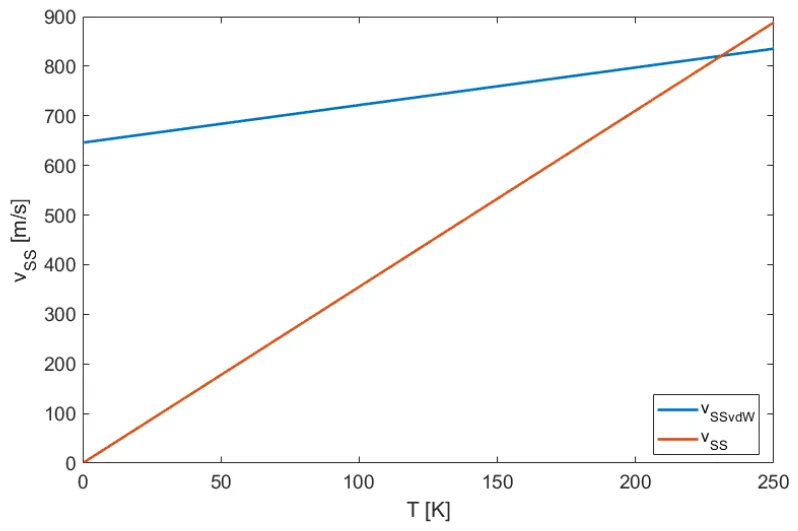
Temperature dependence of the elementary sliding velocity, calculated using Equations (9) and (18). The model assumes one molecule water contaminant layer with constants $a_{2}=1.96\times 10^{-39}\;J\;m^{2}$ and $b_{2}=0.1310\;nm^{2}$ on an elementary surface area of $A_{0}=0.1665\;nm^{2}$.

**Figure 3:**
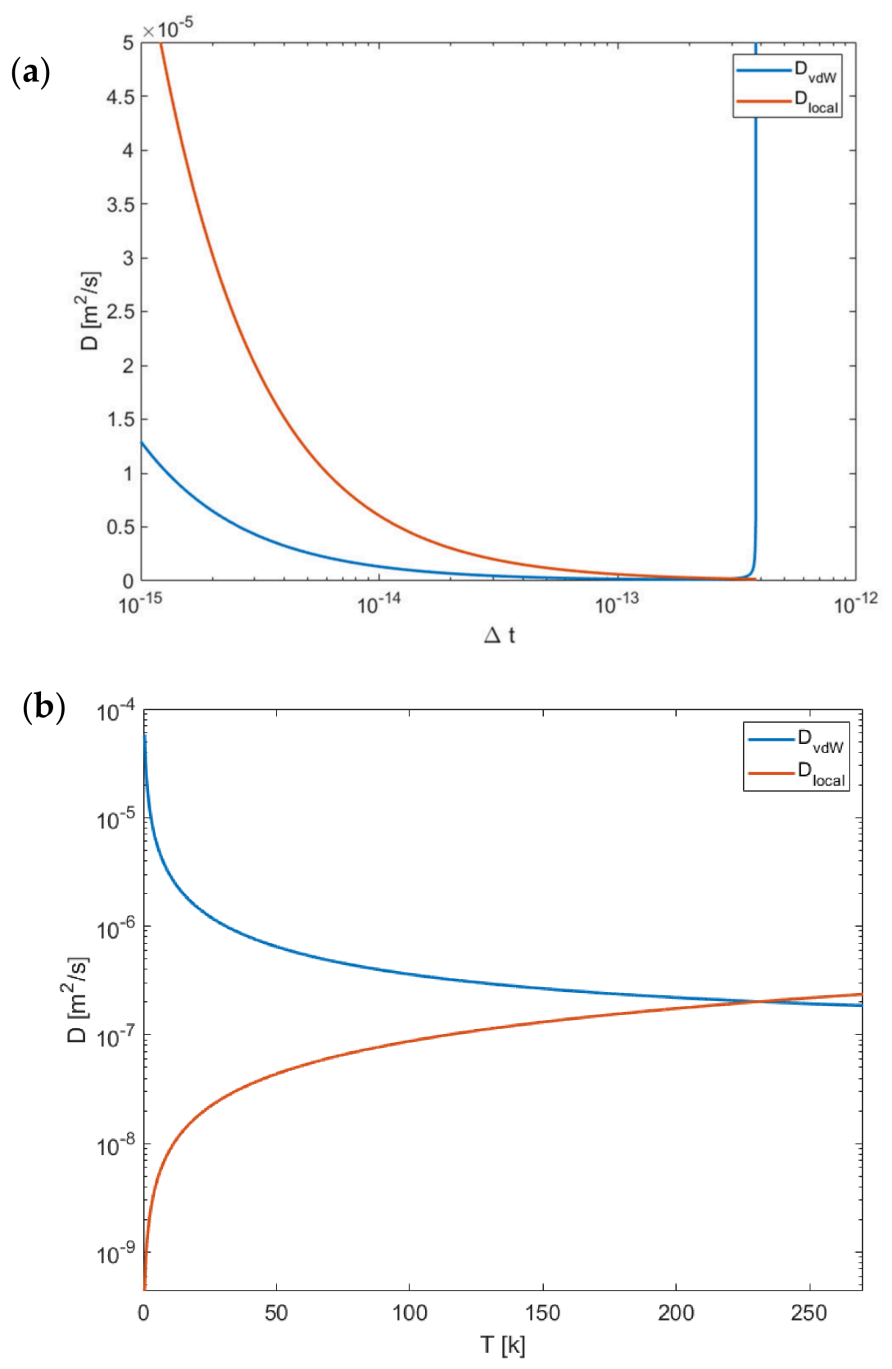
Diffusion coefficient depending on the lubrication time (**a**) and on temperature (**b**). The graphs compare the outcomes of Equation (16) (red line, local reference) and Equation (17) (blue line, contaminated/phonon-mediated). Existence of maximal time of interaction ∆t in limit of temperature goes to zero T→0 due to the rise in (**a**). The system parameters remain identical to those used in Figure 2.

## Data Availability

The original contributions presented in this study are included in the article. Further inquiries can be directed to the corresponding author(s).

## Abbreviations

The following abbreviations are used in this manuscript:

MNET	Mesoscopic Nonequilibrium Thermodynamics
COF	Coefficient of Friction
AFM	Atomic Force Microscopy
FFM	Friction Force Microscopy
vdW	van der Waals
